# Alexithymia and automatic processing of facial emotions: behavioral and neural findings

**DOI:** 10.1186/s12868-020-00572-6

**Published:** 2020-05-29

**Authors:** Nicole Rosenberg, Klas Ihme, Vladimir Lichev, Julia Sacher, Michael Rufer, Hans Jörgen Grabe, Harald Kugel, André Pampel, Jöran Lepsien, Anette Kersting, Arno Villringer, Thomas Suslow

**Affiliations:** 1 Department of Psychosomatic Medicine and Psychotherapy, University of Leipzig, Semmelweisstrasse 10, 04103 Leipzig, Germany; 2 Institute of Transportation Systems, German Aerospace Center, Lilienthalplatz 7, 38108 Brunswick, Germany; 3 Department of Neurology, Max-Planck-Institute of Human Cognitive and Brain Sciences, Stephanstraße 1, 04103 Leipzig, Germany; 4 Clinic of Cognitive Neurology, University of Leipzig, Liebigstrasse 18, 04103 Leipzig, Germany; 5 Department of Psychiatry, Psychotherapy and Psychosomatics, University Hospital Zurich, University of Zurich, Militärstrasse 8, 8021 Zurich, Switzerland; 6 Department of Psychiatry, University Medicine of Greifswald, Ellernholzstraße 1-2, 17475 Greifswald, Germany; 7 Department of Clinical Radiology, University of Münster, Albert-Schweitzer-Campus 1, 48149 Münster, Germany; 8 Nuclear Magnetic Resonance Unit, Max-Planck-Institute of Human Cognitive and Brain Sciences, Stephanstraße 1, 04103 Leipzig, Germany

**Keywords:** Alexithymia, Automatic processing, Priming, Toronto Structured Interview for Alexithymia, Imagination, Fantasizing

## Abstract

**Background:**

Alexithymia is a personality trait characterized by difficulties identifying and describing feelings, an externally oriented style of thinking, and a reduced inclination to imagination. Previous research has shown deficits in the recognition of emotional facial expressions in alexithymia and reductions of brain responsivity to emotional stimuli. Using an affective priming paradigm, we investigated automatic perception of facial emotions as a function of alexithymia at the behavioral and neural level. In addition to self-report scales, we applied an interview to assess alexithymic tendencies.

**Results:**

During 3 T fMRI scanning, 49 healthy individuals judged valence of neutral faces preceded by briefly shown happy, angry, fearful, and neutral facial expressions. Alexithymia was assessed using the 20-Item Toronto Alexithymia Scale (TAS-20), the Bermond-Vorst Alexithymia Questionnaire (BVAQ) and the Toronto Structured Interview for Alexithymia (TSIA). As expected, only negative correlations were found between alexithymic features and affective priming. The global level of self-reported alexithymia (as assessed by the TAS-20 and the BVAQ) was found to be related to less affective priming owing to angry faces. At the facet level, difficulties identifying feelings, difficulties analyzing feelings, and impoverished fantasy (as measured by the BVAQ) were correlated with reduced affective priming due to angry faces. Difficulties identifying feelings (BVAQ) correlated also with reduced affective priming due to fearful faces and reduced imagination (TSIA) was related to decreased affective priming due to happy faces. There was only one significant correlation between alexithymia dimensions and automatic brain response to masked facial emotions: TAS-20 alexithymia correlated with heightened brain response to masked happy faces in superior and medial frontal areas.

**Conclusions:**

Our behavioral results provide evidence that alexithymic features are related in particular to less sensitivity for covert facial expressions of anger. The perceptual alterations could reflect impaired automatic recognition or integration of social anger signals into judgemental processes and might contribute to the problems in interpersonal relationships associated with alexithymia. Our findings suggest that self-report measures of alexithymia may have an advantage over interview-based tests as research tools in the field of emotion perception at least in samples of healthy individuals characterized by rather low levels of alexithymia.

## Background

Recognizing emotions from faces is one of the most important abilities for successful social functioning. In everyday life we identify emotions, intentions, attitudes, and much more from the facial expression of our counterpart [1]. As these expressions change very quickly, we draw our inferences automatically, without thinking about them [2]. However, there are some conditions where people have problems to do so.

Alexithymia is a personality trait that is characterized by an impaired ability to identify and describe one’s own emotions [3]. Moreover, it includes an externally oriented thinking style and impoverished fantasy. The former refers to a tendency to deal with details of external events rather than subjective feelings and thoughts; the latter describes a lack of affect-related imaginal processes.

Besides difficulty in recognizing one’s own feelings, alexithymia has been associated with deficits in the recognition of emotional facial expressions in others [4]. There is evidence that these deficits are pronounced under suboptimal processing conditions. Parker et al. [5] reported that the difficulty in describing feelings was inversely related to the detection of fearful facial emotions when stimuli were presented with temporal constraints, while there was no relation with alexithymia when the faces were presented for a longer time interval. Further studies revealed associations between alexithymia and poor labeling of briefly presented emotional facial expressions [6, 7].

Other studies tried to elucidate *automatic* emotion processing in alexithymia. A way to capture automatic emotion processing is the use of the affective priming paradigm which goes back to Zajonc [8]. He stated that affective reactions can be elicited with minimal stimulus input, even in the absence of conscious awareness. In the affective priming paradigm, a very briefly presented emotional facial stimulus (*prime*) precedes a neutral stimulus (*target*) that must be evaluated concerning its valence. It was revealed that the target is evaluated more positively when it is preceded by a happy facial stimulus (compared to a neutral one) and more negatively when preceded by an angry facial expression [9]. That means, that barely visible emotional facial expressions have an impact on the evaluation of subsequently presented stimuli [10]. They shift judgments in direction of their valence: compared to neutral expression, happy facial expression elicits more positive evaluations of subsequently shown stimuli, whereas angry facial expression leads to more negative evaluations of subsequently displayed stimuli. This phenomenon of evaluative shifting or affect congruent influence of emotional facial expression on judgments is called *affective priming effect*.

Using the affective priming paradigm, a number of neuroimaging studies failed to find a relation of *behavioral* evaluative shifting with alexithymic traits [11–13]. This might be due to small sample sizes (Ns varied between 21 and 33 participants). All these neuroimaging investigations followed a dimensional or correlational approach of analysis including data evaluations at the subscale or facet level. In contrast, in a behavioral study, Vermeulen et al. [14] found a significant effect of alexithymia on affective priming, namely a negative correlation between alexithymia as measured by the 20-Item Toronto Alexithymia Scale (TAS-20) and affective priming due to angry faces. However, Vermeulen et al. [14] did not assess evaluative shifts in their experiments but a reaction-time based type of affective priming which refers to automatic processes of response facilitation and response inhibition. The latter processes have also been examined as a function of alexithymia in a word-based affective priming experiment [15]. Both affective priming studies [14, 15] used a dimensional approach to analyze the effects of alexithymia on automatic emotion processing. However, in the study of Vermeulen et al. (2006) no analysis of data was conducted at the facet level.

According to a recent review of behavioral studies on automatic emotion processing deficits in alexithymia [16] alexithymic traits are related to decreased involuntary attention allocation to emotional stimuli and decreased efficiency of processing emotion faces. It appears that alexithymic individuals have impairments in the rapid perception of hidden threat-related, i.e., angry and fearful, expressions. Results from a visual ERP study confirm that alexithymia is characterized by deficits in the early appraisal of angry faces: change and novelty are poorly detected in angry expressions [17]. Findings from an investigation on the categorization of nonverbal emotional vocalizations suggest that alexithymic deficits in emotion perception could occur across different sensory modalities [18].

Recent reviews and meta-analyses of neuroimaging research investigating the neural correlates of alexithymia provide evidence that alexithymia is associated with a diminished response of the amygdala, suggesting reduced attention to emotion stimuli at a controlled and automatic processing level [19, 20]. Specifically, difficulties identifying feelings appear to be associated with reduced neural activity in the emotional attention system comprising the amygdala, fusiform and occipital gyrus [20]. It was also observed that negative stimuli elicit decreased activation in motor, premotor and cerebellar brain areas, which might account for poor empathic abilities associated with alexithymia. Positive stimuli were found to elicit decreased activation in the insula and precuneus, pointing to reduced emotional awareness in alexithymia regarding positive emotions [19].

There are some fMRI studies in which brain response to masked facial emotions was investigated as a function of alexithymia [11–13, 21]. The last-mentioned investigations used dimensional analyses to investigate the effect of the alexithymia facets. It emerged that alexithymic individuals show a low responsivity to barely visible sad facial expression in brain regions responsible for affective response, encoding, and appraisal, i.e. amygdala, insula, and occipito-temporal areas [11–13]. These regions are known to be involved in the non-conscious processing of emotional contents [22]. Specifically, problems identifying feelings seem to be related to low reactivity of the amygdala to sad faces [13]. Alexithymia was also found to be related to low automatic brain reactivity to happy and surprised facial expression [12, 21]. Moreover, alexithymic individuals have been found to show deficits in early visual processing of fearful body postures [23] and decreased electrodermal response to negative images at an automatic processing level [24]. The latter two studies used categorical analyses with group comparisons of low and high alexithymic individuals to investigate the effects of alexithymia on automatic emotion processing. Interestingly, Pollatos et al. [24] observed that the effect of alexithymia on electrodermal response to negative pictures was driven by the alexithymia facets difficulties in identifying and verbalizing emotions.

Against this background, it can be assumed that alexithymia might be due to low responsiveness of the amygdala and other brain structures to hidden emotional information. The effortless production of responses in basic emotion processing systems of the brain seems to give essential input to higher processing systems. When fundamental processes of perception and affective response are impaired, differentiation and recognition of emotional reactions might become difficult [12, 16]. The evidence stemming from the neuroimaging studies is, in general, in line with the behavioral data and with conclusions drawn in a recent review of physiological reactivity to emotions in alexithymia [25]. According to this review, autonomic reactivity to a wide array of affective stimuli is reduced in alexithymic individuals. Specifically, skin conductance response to emotional cues seems to be decreased in alexithymia suggesting deficits in the spontaneous mobilization of resources to initiate action [25]. Taken together, the present literature suggests an automatic hyporesponsiveness to emotional information in alexithymia at a behavioral, physiological, and brain response level.

In previous studies, alexithymic features have been predominantly assessed by self-report (e.g., the TAS-20 [26]), although alternative measures have been recommended. The TAS-20 has been criticized repeatedly for measuring anxious and depressive tendencies [27, 28]. Furthermore, it does not assess impoverished fantasy and thus lacks an important feature of alexithymia [29]. In contrast, the Bermond-Vorst Alexithymia Questionnaire (BVAQ [30]) is a self-report measure that includes a fantasy scale (Fantasizing) and items regarding the emotional reactivity component of alexithymia (Emotionalizing), both of which are thought to be deficient in alexithymic individuals. However, it is controversial whether emotional reactivity can be considered part of the alexithymia construct. A network analysis based on data from a large sample failed to support emotionalizing as a distinct component of the construct [31].

An alternative way to assess alexithymic tendencies is by observer-rating. The Toronto Structured Interview for Alexithymia (TSIA [32]) measures alexithymia through the ratings of an interviewer that are formed by including examples from the respondent. This procedure might lead to a more comprehensive and valid evaluation, because it is not biased by the self-evaluation of the alexithymic individuals. Like the BVAQ, the TSIA comprises items regarding impoverished fantasy.

The aim of this study was to investigate automatic emotion processing in alexithymia by adopting the affective priming paradigm while subjects underwent functional neuroimaging. For this purpose, we followed a dimensional approach of analysis and did not compare groups based on cut-off scores. Notably, this is the first study on alexithymia and automatic emotion processing in which the TSIA, an interview-based measure, was administered to assess alexithymic tendencies, additionally to self-report measures. Against the background of previous findings, a negative relationship was expected between alexithymia and automatic evaluative shifting due to emotional faces, i.e. behavioral affective priming. Moreover, it was hypothesized that alexithymia is negatively correlated with neural activation of the amygdala and other brain regions during the processing of masked emotional faces.

## Methods

### Participants

Fifty-two healthy subjects aged between 18 and 29 years participated in the study. Participants were recruited via public notices and online advertisements. The notices were posted in public places such as libraries and student residencies. All of them were right-handed, native speakers of German and had normal or corrected-to-normal visual acuity. None of them had a psychiatric disorder in present or past as assessed by the Structured Clinical Interview for DSM-IV [33]. Yarkoni and Braver [34] proposed to examine at least 40 participants for correlational analyses in neuroimaging research on individual differences in cognition. All participants gave written informed consent and received financial compensation. The procedure of the study was approved by the ethics committee of the Medical School at the University of Leipzig and was in accordance with the Declaration of Helsinki. Three participants had to be excluded from further analysis, one because of high depression score (BDI-II > 14) at time of scanning, one because of excessive head motion in the scanner (> 3 mm translation), and one because of high deviation in his behavioral reactions (evaluative shifts > 3 SD from mean). Thus, the final sample consisted of 49 participants (23 female, mean age = 23.3 years, SD = 2.8).

### Assessment of alexithymia and control variables

Alexithymia was assessed by three different measures: two self-report questionnaires, the 20-Item Toronto Alexithymia Scale (TAS-20; German version [35] and the Bermond-Vorst Alexithymia Questionnaire (BVAQ; German version [36]) as well as one observer-rated measure, the Toronto Structured Interview for Alexithymia (TSIA; German version [37]).

The TAS-20 comprises three factors, difficulty identifying feelings (DIF), difficulty describing feelings (DDF) and externally oriented thinking (EOT). Answers are rated on a 5-point Likert scale (range of possible scores: 20–100) with higher scores indicating higher alexithymia.

The BVAQ is a 40-item scale comprising five subscales: Identifying, Verbalizing, Analyzing, Emotionalizing, and Fantasizing. Ratings are made on a 5-point Likert scale (range of possible scores: 40-200) with higher scores indicating higher alexithymia.

The TSIA is an observer-rated, interview-based measure consisting of 24 questions relating to the factors difficulty identifying feelings (DIF), difficulty describing feelings (DDF), externally oriented thinking (EOT), and imaginal processes (IMP). The answers are rated on a three-point scale; total scores range from 0 to 48 with higher scores indicating higher alexithymia. The TSIA was administered by one trained interviewer (NR) who also did the ratings. The interviewer was trained in the administration and the scoring of the interview by one of the translators of the German version of the TSIA, with discussions of the use of the prompts and probes and the scoring guidelines. At the time of rating, she was not informed about TAS-20 and BVAQ scores of the participants. Interviews were conducted individually and took about 90 min.

To control for negative affectivity (NA), we assessed depressive symptoms by the Beck Depression Inventory (BDI-II; German version [38]) as well as trait anxiety using the trait version of the State-Trait Anxiety Inventory (STAI-T; German version [39]). Alexithymia as assessed by the TAS-20 and in particular the component difficulties in identifying feelings have been found to be positively associated with depressive symptoms and trait anxiety [40, 41]. Control of trait anxiety appears especially important in a study focusing on the perception of threat-related stimuli [42].

Alexithymia measurement was done before, measurement of NA after the MRI-session.

### fMRI experiment and procedure

Facial stimuli consisted of gray-scale normalized photographs of happy (HA), angry (AN), fearful (FE), and neutral (NE) expressions of ten different individuals (five female) [43]. Neutral and emotional expressions were administered as primes. Angry and fearful faces were used as negative prime stimuli in our study since in a recent review [16] it was concluded that alexithymia and its facets difficulties identifying feelings and externally oriented thinking are linked in particular to impairments in the automatic processing of *threat*-*related* facial stimuli, i.e. *directly* threatening or angry faces and *indirectly* threatening or fearful faces. Happy faces were presented as primes to investigate automatic processing of *positive* facial expressions. Faces expressing happiness and anger have already been shown as primes in the first affective priming studies [9, 44]. Faces with neutral expressions were administered as primes to define the evaluative baseline condition. Neutral expressions of the same individuals were used as masking stimuli. To avoid identity of prime and masking stimuli in the neutral prime condition we applied vertically mirrored faces as masks. The experiment comprised 80 trials (20 trials per prime condition). Each trial lasted for 9 s, starting with the presentation of a fixation cross in the center of the screen for 800 ms followed by the prime for 33 ms, which was followed by a neutral face for 467 ms. Then, a blank screen with no stimulus appeared for 7.7 s. In this time period participants were asked to evaluate the neutral mask face as expressing rather negative or rather positive feelings by button press (− 1.50, − 0.50, + 0.50, + 1.50). The prime presentation duration was set at 33 ms since this duration has proven to elicit affective priming effects in previous studies [45–47].

Participants had one response pad per hand with two buttons each for responses with the index and middle fingers. During the response window, the order of attribution was presented on the bottom of the screen (e.g., ‘− 1.50’ leftmost meaning the left middle finger indicates the most negative evaluation). One half of the sample gave negative evaluations with the left pad, the other half with the right pad. Participants were instructed to respond quickly and intuitively. Trials were presented in one of two fixed random sequences with constriction of no repetition of an individual and no more than one repetition of a prime condition on consecutive trials. An LCD-Projector (Sanyo PLC-XP50L) was used as stimulation device. It is equipped with special objective lenses enabling to present small images on long distances. Presentation of stimuli was enabled via back-projection onto a screen positioned at the rear-end of the bore. The screen could be viewed via a mirror mounted on the head-coil. The experiment was performed using Presentation software (Neurobehavioral Systems, Inc., Berkeley, CA, http://www.neurobs.com) installed on a Dell Latitude E6540.

### Analysis of psychometric and behavioral data

For alexithymia, we calculated descriptive statistics and intercorrelations for all scales of the different alexithymia measures. To control for NA, we correlated alexithymia scores with the BDI-II and STAI-T.

Regarding behavioral performance, we calculated the evaluative responses (by averaging the evaluative responses per condition) as well as priming scores (by subtracting the mean evaluative response for neutral from the mean evaluative response for each emotional condition). In this way, we got a measure for the mean evaluative rating (for happy, angry, fearful, and neutral) and a measure for the behavioral evaluative shifting (i.e., affective priming; owing to happy, angry, and fearful facial expressions). To test for differences between evaluation scores, we calculated a repeated measures analysis of variance (ANOVA) with one within-subjects factor (prime condition: happy, angry, fearful and neutral). Finally, correlations were calculated between priming scores and alexithymia measures. To keep the interpretation of the data simple, we inverted the negative priming scores (for the conditions angry and fearful primes) before doing correlation analyses. That means, positive priming values suggest in all cases prime-valence congruent evaluative shifting regardless of emotional quality of the prime (happy, angry, or fearful). For example, a positive priming score for angry faces indicates that the evaluations of the target face were more negative in the angry prime condition compared to the neutral prime condition and a positive priming score for happy faces indicates that the evaluations of the target face were more positive in the happy prime condition compared to the neutral prime condition.

### fMRI data acquisition and data analysis

Participants underwent structural and functional MRI scanning on a 3 T scanner (Magnetom Verio, Siemens, Erlangen, Germany) using a standard 12-channel head coil. For anatomical registration, high resolution T1-weighted 3D MP-RAGE [48] structural images were obtained. Magnetization preparation consisted of a non-selective inversion pulse. The imaging parameters were TI = 650 ms, TR = 1300 ms, TE = 3.5 ms, flip angle = 10°, isotropic spatial resolution of 1 mm x 1 mm x 1 mm, two averages. Blood oxygen level-dependent sensitive images were obtained, entailing a T2*—weighted echo-planar imaging (EPI) sequence (matrix 64^2^; resolution 3 mm × 3 mm × 4 mm; gap 0.8 mm; TR = 2 s; TE = 30 ms; flip angle 90°; 30 slices; interleaved slice acquisition; 400 volumes). Slices were oriented parallel to the anterior–posterior commissure plane.

MRI data were preprocessed and analyzed using SPM8 (http://www.fil.ion.ucl.ac.uk/spm/). The initial five volumes, acquired before starting the paradigm, were discarded to allow longitudinal magnetization to reach equilibrium. Functional volumes were slice-time corrected (temporal middle slice as reference), spatially realigned to the first image and corrected for movement-induced image distortions (six-parameter rigid body affine realignment). Functional and anatomical images were co-registered. Anatomical images were then segmented, including normalization to a standard stereotaxic space (Montreal Neurological Institute, MNI). The normalization parameters were then applied to the functional EPI images. The resulting voxel size for the functional images was 3 mm × 3 mm × 3 mm. A temporal high-pass filter (128 s) was applied to remove low-frequency noise. For the functional data, spatial smoothing was performed using a three-dimensional Gaussian filter of 6 mm full-width at half-maximum.

Data were analyzed using an event-related design by modeling the onset of the primes and by convolving this function with the hemodynamic response function for the different emotional conditions. First-level t-contrasts were calculated for each subject by contrasting each emotional condition with the neutral one (HA > NE, AN > NE, FE > NE). These contrast images were then entered in the second-level random-effects models for main effects. To investigate the influence of alexithymia, we calculated regression models with the different alexithymia scales as regressors for each emotional condition. Further, we calculated regression models with individual priming scores as regressors for each emotional condition. For the main effects, significance was tested against a family-wise error (FWE)—corrected threshold at *p* < 0.05. For the regression models, results were considered significant if they survived FWE correction with the cluster-level threshold *p*_FWE_ < 0.05 and an initial voxel threshold *p* < 0.001.

Furthermore, we calculated region of interest (ROI) analyses focusing on the amygdala as a key structure for emotion processing. The amygdala volume was based on the Wake Forest University (WFU) pickatlas [49]. For ROI analyses, we applied a significance threshold of *p*_FWE_ < 0.05 and an initial voxel threshold *p* < 0.001.

### Detection task

Outside the scanner after the fMRI experiment, participants performed a detection task to control for detection of the primes. The task consisted of 40 trials, 10 per prime condition. Stimulus presentation was the same as in the fMRI experiment, that is, prime stimuli were presented for 33 ms and subsequently masked for 467 ms with a neutral expression from the same individual. Participants had to label the expression of the prime using a forced-choice procedure. That is, they had to indicate if the presented facial expression was a happy, angry, fearful, sad, or neutral emotion (although sad faces were not presented). The non-parametric index of sensitivity A’ [50] was used to assess detection performance. This index is an extension of the Grier sensitivity index [51] and is appropriate for cases when the false alarm rate exceeds the hit rate. The A’ index takes into account hit and false alarm rates. Chance performance is indicated at A’ = 0.5. To rule out the possibility that detection of primes has an influence on prime evaluation, we correlated detection scores with priming scores for each prime condition.

## Results

### Alexithymia measures

#### Descriptive statistics

Mean scores, mean item scores, standard deviations, and ranges for the scales of TAS-20, BVAQ, and TSIA are shown in Table 1. Intercorrelations of alexithymia scales are available as supplement (see Additional file 1: Table S1).

**Table 1:** Descriptive statistics for the alexithymia measures

Scale	Mean	SD	Range	Mean item score^a^
TAS-20 total	43.20	10.70	22–71	2.16
TAS-DIF	12.49	4.29	7–25	1.78
TAS-DDF	12.45	4.59	5–24	2.49
TAS-EOT	18.27	4.59	10–31	2.28
BVAQ total	106.44	24.01	65–166	2.66
Identifying	18.76	5.73	8–36	2.34
Verbalizing	22.94	7.82	9–39	2.87
Analyzing	18.76	6.55	9–32	2.34
Emotionalizing	20.16	6.32	11–35	2.52
Fantasizing	25.84	7.29	13–37	3.23
TSIA total	16.59	9.82	2–37	0.69
TSIA-DIF	1.35	2.05	0–8	0.22
TSIA-DDF	2.73	3.37	0–11	0.46
TSIA-EOT	5.80	3.38	0–12	0.97
TSIA-IMP	6.71	2.92	1–11	1.12

^a^Rating scale from 1 to 5 for the TAS-20 and BVAQ and from 0 to 2 for the TSIA

N = 49. *TAS-20* 20-Item Toronto Alexithymia Scale, *DIF* difficulties identifying feelings, *DDF* difficulties describing feelings, *EOT* externally oriented thinking, *BVAQ* Bermond-Vorst Alexithymia Questionnaire, *TSIA* Toronto Structured Interview for Alexithymia, *IMP* imaginal processes

There was no significant correlation (all *p*s > 0.1) between the alexithymia scales and the measures of negative affectivity (BDI-II and STAI-T), except for the BVAQ-subscale Verbalizing, which was positively correlated with STAI-T (see Table 2). To take into account possible influences of trait anxiety, we included STAI-T scores as a control variable in further analyses concerning Verbalizing. As this did not change the results substantially, we do not present them here.

**Table 2:** Correlations between alexithymia scales and measures of negative affectivity

Scale	Level of depressive symptoms (BDI-II)	Trait anxiety (STAI-T)
TAS-20 total	0.00	0.13
TAS-DIF	− 0.09	0.15
TAS-DDF	0.13	0.17
TAS-EOT	− 0.04	− 0.02
BVAQ total	0.12	0.10
Identifying	0.01	0.16
Verbalizing	0.19	0.30*
Analyzing	0.04	0.11
Emotionalizing	0.02	− 0.07
Fantasizing	0.11	− 0.15
TSIA total	− 0.10	− 0.05
TSIA-DIF	− 0.15	− 0.01
TSIA-DDF	0.03	0.12
TSIA-EOT	− 0.15	− 0.09
TSIA-IMP	− 0.09	− 0.20

* *p* < 0.05

N = 49. *TAS-20* 20-Item Toronto Alexithymia Scale, *DIF* difficulties identifying feelings, *DDF* difficulties describing feelings, *EOT* externally oriented thinking, BVAQ Bermond-Vorst Alexithymia Questionnaire, *TSIA* Toronto Structured Interview for Alexithymia, *IMP* imaginal processes, *BDI-II* Beck Depression Inventory, *STAI-T* State-Trait Anxiety Inventory—trait version

#### Behavioral performance

Means and standard deviations of evaluative responses and priming scores are shown in Table 3. Overall, there was a tendency to judge faces rather negative. To test for differences between evaluation scores, we calculated a repeated measures analysis of variance (ANOVA) with one within-subjects factor (prime condition: happy, angry, fearful, and neutral). There was no significant effect of prime condition on evaluation scores (*F*(3144) = 0.61, *p* = 0.61).

**Table 3:** Evaluative responses and priming scores based on happy, angry, and fearful facial expressions (baseline: neutral faces)

Prime condition	Evaluative responses		Priming scores	
	Mean	SD	Mean	SD
Happy	− 0.070	0.259	0.012	0.150
Angry	− 0.097	0.254	− 0.015	0.131
Fearful	− 0.085	0.254	− 0.003	0.124
Neutral	− 0.082	0.261	–	–

N = 49

To examine if there are differences in affective priming as a function of alexithymia, we correlated the priming scores for happy, angry, and fearful primes with the different alexithymia measures (Table 4). Results showed significant correlations across alexithymia measures in the expected negative direction. That is, heightened alexithymic characteristics went along with less valence-congruent shifts due to happy, angry, and fearful primes. Specifically, TAS-20 total score and BVAQ total score were significantly negatively correlated with the priming score for angry faces. Moreover, the BVAQ subscales Identifying, Analyzing, and Fantasizing showed significant negative correlations with priming based on angry faces (see Table 4). Identifying (BVAQ) was also negatively related to affective priming based on fearful faces. For the TSIA, there was a significant negative correlation between Imaginal processes and affective priming due to happy faces.

**Table 4:** Correlations between alexithymia scales and affective priming scores based on happy, angry, and fearful facial expressions

	Prime condition		
	Happy	Angry	Fearful
TAS-20 total	0.19	− 0.30*	− 0.18
TAS-DIF	0.22	− 0.23	− 0.28
TAS-DDF	0.18	− 0.27	− 0.21
TAS-EOT	0.07	− 0.21	0.04
BVAQ total	0.03	− 0.35*	− 0.17
Identifying	0.25	− 0.29*	− 0.34*
Verbalizing	0.07	− 0.26	− 0.14
Analyzing	0.03	− 0.36*	− 0.07
Emotionalizing	− 0.21	− 0.05	0.01
Fantasizing	− 0.01	− 0.29*	− 0.09
TSIA total	− 0.22	− 0.11	− 0.02
TSIA-DIF	− 0.01	− 0.09	− 0.15
TSIA-DDF	− 0.10	− 0.11	− 0.08
TSIA-EOT	− 0.22	− 0.12	0.02
TSIA-IMP	− 0.38**	− 0.04	0.09

* Significant at *p* < 0.05 (two-tailed), ** significant at *p* < 0.01 (two-tailed)

N = 49. *TAS-20* 20-Item Toronto Alexithymia Scale, *DIF* difficulties identifying feelings, *DDF* difficulties describing feelings, *EOT* externally oriented thinking, *BVAQ* Bermond-Vorst Alexithymia Questionnaire, *TSIA* Toronto Structured Interview for Alexithymia, *IMP* imaginal processes

#### Detection task performance

Sensitivity values A’ were as follows: for happy faces 0.76 (*SE* = 0.01), for angry faces 0.51 (*SE* = 0.03), for fearful faces 0.60 (*SE* = 0.03), and for neutral faces 0.65 (*SE* = 0.02). For happy, fearful, and neutral faces, these values were significantly different from 0.5 (*p* < 0.05). For angry faces, the sensitivity value did not differ significantly from 0.5 (*p* > 0.05). Thus, happy, fearful, and neutral faces have been detected above chance-level in the detection task. However, detection was still far from perfect recognition.

To ensure that possible detection of primes (especially happy primes) had no influence on evaluative ratings, we correlated the sensitivity values from the detection task with the relevant priming scores (e.g., sensitivity value for happy faces with priming score for happy faces). All correlations were non-significant (*r* = 0.08, *p* = 0.58 for happy; *r* = − 0.05, *p* = 0.74 for angry; *r* = − 0.08, *p* = 0.61 for fearful). Against this background, it can be concluded that detection of prime faces had no systematic influence on behavioral evaluative shifts.

#### Neuroimaging results

##### Main effects of emotional faces on brain activation: whole-brain analysis

Happy versus neutral primes elicited significant activation in three clusters. One cluster was located in the left superior temporal gyrus (STG), extending to supramarginal gyrus (Montreal Neurological Institute (MNI) coordinates xyz = [− 54 − 49 13], cluster extent k = 50, *p*_FWE_ < 0.001). A second cluster was found in the right inferior parietal lobule, extending to supramarginal gyrus (xyz [63 − 43 25], k = 10, *p*_FWE_ < 0.001). Finally, there was a significantly activated cluster in the right STG, extending to the middle temporal gyrus (xyz = [48 − 37 10], k = 89, *p*_FWE_ ≤ 0.001). In our whole-brain analyses, no main effects were revealed for angry and fearful faces.

##### Main effects of emotional faces on amygdala activation: ROI analysis

There was no significant amygdala activation revealed in response to happy, angry, and fearful primes (compared to neutral primes).

##### Correlations of alexithymia with brain activation: whole-brain analysis

A significant cluster was found indicating a positive correlation between the TAS-20 total score and activation of the superior frontal gyrus, extending to the medial frontal gyrus in response to happy primes (HA > NE) (xyz [21 44 19], k = 56, *p*_FWE-cluster_ < 0.05). No significant clusters were revealed for other contrasts or other alexithymia scales.

##### Correlations of alexithymia with amygdala response: ROI analyses

The ROI analyses of the amygdalae revealed no significant correlation between alexithymia scales and amygdala reactivity to happy, angry, or fearful faces.

##### Correlations of priming scores with brain activation: whole-brain analysis

Whole-brain analyses with individual priming scores as regressors showed no significant correlation of clusters activated in response to happy, angry, or fearful faces with priming scores.

##### Correlations of priming scores with amygdala response: ROI analyses

The ROI analyses of the amygdalae revealed no significant correlation between individual priming scores and amygdala reactivity to happy, angry, or fearful faces.

## Discussion

In the present study, we investigated automatic processing of emotional facial expressions as a function of the personality trait alexithymia. In addition to self-report questionnaires, the TAS-20 and the BVAQ, we employed an interview-based measure, the TSIA, to assess alexithymic tendencies. An affective priming paradigm was administered to examine automatic processes of emotion perception. In our study, evidence was obtained that individual differences in alexithymic characteristics are associated with priming performance (i.e., evaluative shifts) and, to much lower extent, with altered brain reactivity to masked facial emotions. At the behavioral level, we found primarily correlations for the self-report alexithymia measures (TAS-20 and BVAQ) with affective priming indices. In contrast, at the neural level, we observed only one correlation between alexithymia measures and automatic brain activity in response to masked facial emotions: the TAS-20 total score was positively related to brain response to masked happy faces.

In our study, we found negative correlations of medium size between several self-report alexithymia facets and affective priming based on angry faces. That is, individuals with heightened alexithymia scores showed less valence-congruent shifting due to masked angry facial expressions. Thus, the number of significant correlations varied substantially depending on the test instrument, alexithymia facet, and type of prime. Five out of the seven correlations observed were related to affective priming due to angry faces. There was only one correlation of alexithymia with affective priming based on fearful faces and one correlation with affective priming based on happy faces. This suggests, that alexithymic features are most robustly associated with the automatic processing of facial anger. Interestingly, results from a visual ERP study suggest that perception of anger in faces is disrupted in alexithymia [17]. There are also findings from other behavioral, electromyographic and neuroimaging studies on alexithymia showing deficits in response to anger. Greater levels of alexithymia have been found to be related to attenuated cardiovascular reactivity to angry events [52]. This result provides evidence for a hypoarousal model of alexithymia during anger provoking events. Furthermore, alexithymic individuals showed less corrugator-muscle imitation in response to angry faces [53]. Thus, it appears that a form of primitive catching of other persons’ anger by automatically mimicking and synchronizing their facial expressions is decreased in alexithymia. A hyporesponsiveness of the basal ganglia, insula and anterior cingulate cortex to angry facial expressions has been documented in alexithymic individuals which may contribute to their dysfunction in recognizing anger in faces [54, 55].

As there were no significant correlations of sensitivity indices with priming scores, our data indicate that affective priming was not systematically influenced by the awareness of primes. Our results are in line with those of Vermeulen et al.’s study [14] in which a negative relation of self-reported alexithymia with affective priming due to angry faces was revealed. However, as Vermeulen et al. [14] used a different affective priming approach which assessed response facilitation and inhibition, the present study is the first showing a significant relation between alexithymia and *automatic evaluative shifting* due to angry primes. The study of Vermeulen et al. [14] can be criticized for not analyzing the data at the facet level. Concerning our results, it must be emphasized that both total scores of the self-report alexithymia measures exhibited significant negative correlations with affective priming based on angry faces but were not related to priming based on happy or fearful primes. This means, that the global level of self-reported alexithymia characteristics was found to be associated with less affective priming owing to angry facial expressions. In contrast to the TAS-20, the BVAQ showed several correlations of alexithymia facets with affective priming scores. The BVAQ subscales Identifying, Analyzing, and Fantasizing were all negatively associated with affective priming due to angry faces. Importantly, in a recent review on alexithymia and automatic emotion perception in which the results of twelve behavioral studies (administering different experimental methods such as emotional Stroop tasks, emotional visual remapping of touch, chimeric faces tasks, attentional blink and priming tasks) were analyzed, it was concluded that especially the alexithymia facets difficulties identifying feelings and externally oriented thinking are related to impairments in the automatic processing of threat-related facial stimuli, i.e. angry and fearful faces [16]. Our data fit in well with this observation considering that the BVAQ subscale Identifying was found to be also negatively associated with affective priming due to fearful faces.

Our behavioral findings indicate that persons with heightened alexithymia could be primarily impaired in recognizing anger of briefly shown, unattended facial expressions and/or in integrating this information into subsequent explicit conclusions and judgments. It appears that alexithymia is characterized by less efficient reading out and use of minimal anger information in the conscious appraisal of social stimuli. Such deficits in sensitivity for subtle socio-emotional signals might contribute to less emotional attunement of individuals with heightened alexithymia within interpersonal relationships [56].

Angry faces occur in situations where social rules and expectations have been violated [57]. More specifically, expressions of anger signal that a person experiences an obstruction of his/her goals and at the same time is convinced to have the capacity to cope with the situation and to assert his/her interests [58]. Healthy individuals manifest automatically adaptive defensive responses such as physiological signs of freezing to angry faces [59]. At the motor level, it appears that angry facial expressions potentiate approach-motivation in order to confront and resolve social challenges [60]. It can be assumed that the recognition of interpersonal anger signals is a prerequisite for these adaptive reactions. People who take no notice of violations of others’ interests and goals should be perceived as ignorant and selfish. It is known that alexithymic persons have a tendency to communicate with others in an unempathic, cold, or detached way [61]. Failure to recognize anger in the face of others should deprive alexithymic individuals of important information that their own behavior was inappropriate and that corrective action is needed. It can be summarized that one mechanism underlying interpersonal problems in alexithymia could be a poor sensitivity for facial expressions of anger.

Our data suggest that difficulties in identifying and analyzing feelings go along with problems in recognizing hidden facial expression of anger. Interestingly, externally oriented thinking style was correlated with reduced speed and efficiency of processing threatening facial emotions in an attention blink study [62]. Furthermore, in an experiment based on the emotional Stroop task it was found that individuals characterized by externally oriented thinking manifest reduced involuntary attention allocation to emotional lexical stimuli [63]. Thus, it appears that externally oriented thinking is linked to impairments in rapid emotion processing. Individuals with heightened alexithymia might need more perceptual information to identify threat-related facial expressions [64].

The BVAQ has hitherto been used less frequently in alexithymia research in comparison with the TAS-20. For example, only in one out of twelve behavioral studies on alexithymia and automatic emotion perception the BVAQ was administered as alexithymia measure whereas the TAS-20 was applied in all twelve studies [16]. Overall, at the behavioral level it can be noted that there is a substantial consistency of correlation results across the self-report measures, BVAQ and TAS-20. Still, the present data indicate that the BVAQ could be a more useful test instrument than the TAS-20 to reveal relationships of alexithymia with automatic perception parameters at the subscale level. This could be due to the fact that the BVAQ includes more items and more subscales than the TAS-20. In our study, the mean item scores and the variability of scores (standard deviations) were descriptively higher for the BVAQ than the TAS-20 which might facilitate detection of relationships with emotion perception characteristics. The BVAQ has also the advantage to include a subscale assessing impoverished fantasy which the TAS-20 does not. Because in the majority of studies examining alexithymia and emotion processing the TAS-20 has been used relations with the alexithymia facet imagination could not be revealed. Our findings indicate instead that it could be valuable to consider and assess reduced imagination in future neurocognitive research on alexithymia. In the present study, the BVAQ scale Fantasizing and the TSIA scale Imaginal processes correlated both negatively with an affective priming score.

It is noteworthy that the TSIA as an essentially promising but also time-consuming interview measure of alexithymia showed only one correlation with affective priming scores. This disappointing outcome may be due to the fact that many of our healthy study participants had relatively low alexithymia scores. Interestingly, in previous studies on alexithymia and emotion perception in which interview-based or observer-rated measures were administered to healthy individuals self-reported alexithymia was also found to be a better predictor of emotion processing indices than the scores derived from interview or observer rating [6, 65]. Taken together, these findings suggest that self-report measures of alexithymia may have an advantage over interview-based or observer-based tests as research tools in the field of emotion perception at least in samples of healthy individuals. The TSIA might be worse in differentiating between individuals with rather low levels of alexithymia because of a less fine-grained scale (3-point-Likert-scale (0 to 2) compared to the 5-point scale of the BVAQ and the TAS-20). The TSIA could be a better predictor in samples including highly alexithymic individuals as these persons appear less able to correctly assess their own difficulties in differentiating and describing emotions.

With regard to brain activity as a function of alexithymia, we found only one correlation between alexithymia measures and neural response to masked facial emotions: the TAS-20 total score was positively correlated with the activity in the superior and medial frontal gyrus in response to masked happy faces. That is, high alexithymic individuals appear to exhibit a stronger automatic brain response to positive facial expressions in some prefrontal cortical areas. Based on these results, our hypothesis of a negative relation between alexithymic features and brain response could not be confirmed. One possible explanation for the observed positive correlation of alexithymia with activation of prefrontal areas might be that individuals with heightened alexithymia have more difficulties in making evaluative decisions than low alexithymic individuals. Thus, the stronger activation might reflect a mechanism to compensate for deficits in emotion appraisal. Frewen et al. [66] observed in a sample of healthy individuals that higher levels of alexithymia were associated with increased activation in the medial prefrontal cortex during imagination. In another neuroimaging study [67], men with alexithymia demonstrated more activation in the mediofrontal cortex, and middle frontal gyrus during the perception of positive images than men without alexithymia. It appears that alexithymia could be linked to structures involved in the appraisal of the emotional content of environmental stimuli. The medial prefrontal gyrus has been shown to be involved in the representation of stimulus value [68]. Medial prefrontal areas appear to be not only involved in explicit evaluation processes but can also be activated during implicit or automatic valuation processes [69]. There is evidence that the ventromedial prefrontal cortex regulates decision making in situations of ambiguity or uncertainty [70]. Thus, it can be argued that the stronger activation of superior and medial frontal regions to masked happy faces might reflect a mechanism to compensate for deficits in emotion appraisal. However, overall, we did not find evidence for robust relationships between alexithymia dimensions and automatic brain response to masked facial emotions in the present study. We revealed only one correlation with brain activity for the TAS-20 but none for the BVAQ and the TSIA.

In our study, no correlations were found between alexithymia measures and automatic brain response to negative (fearful and angry) faces. This is somewhat surprising since in several previous fMRI studies relationships were revealed between alexithymia and low reactivity to barely visible sad facial expression in brain areas involved in appraisal, encoding, and affective response, i.e. amygdala, insula and temporo-occipital areas [11–13]. Differences in findings could be due to more lenient statistical thresholds in the former studies. Against this background, it can be concluded that more research is needed to clarify the link between alexithymia and automatic brain response in general and, specifically, the role of superior and medial frontal gyrus activity having compensatory functions in alexithymia.

It has to be mentioned as a limitation that the mean TAS-20 alexithymia total score (43.2) of the healthy, young participants in our study was relatively low compared to the average score found in the general population in Germany [71]. The variance with such a mean score could be restricted and reduce the magnitude of correlations. However, our TAS-20 total score was comparable to that of healthy, well-educated young adults (41.42) found in a large German sample (n = 241) [41]. Our mean TSIA total score (16.59) was also not very different from the TSIA total score observed in a Swiss sample of psychiatric patients (15.81) [72] or from the TSIA total scores found in a healthy Canadian community sample (women: 17.35; men: 16.38 [32]). Against this background, it might be that the relationships revealed in our study are even stronger in samples showing higher alexithymia scores. Future studies should investigate more heterogeneous and clinical samples adopting the affective priming paradigm.

It must be noted as a further limitation of our study that we excluded all participants with heightened BDI-II score or suspicion of depression. This recruitment procedure could underlie the observed non-correlation between alexithymia scales and negative affectivity (i.e., level of depression and trait-anxiety) which may reduce the generalizability of our findings. However, it has to be pointed out that, according to previous studies, only the alexithymia component difficulties in identifying feelings has been found to be substantially associated with depressive symptoms and trait anxiety [40, 41].

## Conclusion

Taken together, our study revealed new insights into automatic emotion processing in alexithymia. We administered two self-report measures, the TAS-20 and the BVAQ, and an interview-based measure, the TSIA, along with an affective priming task to a sample of healthy, young individuals to investigate for the first time the effects of alexithymia on *automatic evaluative shifts* elicited by masked emotional facial expressions. Evidence was found for associations between alexithymic characteristics and less automatic affective priming based on angry faces. The global level of self-reported alexithymia was found to be related to less affective priming owing to angry facial expressions. At the facet level, difficulties identifying feelings, difficulties analyzing feelings, and impoverished fantasy seem to come along with reduced affective priming due to angry faces. According to our data, alexithymic features are related to less sensitivity for covert facial expression of anger. This perceptual alteration could reflect impaired automatic recognition or integration of social anger signals into judgemental processes and might contribute to the problems in interpersonal relationships associated with alexithymia. Difficulties identifying feelings were related to less priming due to angry and fearful faces. Overall, we did not find evidence for robust relationships between alexithymia dimensions and automatic brain response to masked facial emotions. Alexithymia as assessed by the TAS-20 appears to be associated with heightened brain response to masked positive facial expressions in superior and medial frontal cortical areas which might reflect a mechanism to compensate for deficits in emotion appraisal. Our data indicate that self-report measures of alexithymia may have an advantage over interview-based tests as research tools in the field of emotion perception at least in samples of healthy individuals characterized by rather low levels of alexithymia.

## Supplementary information

**Additional file 1: Table S1.** Correlations between alexithymia measures (N = 49).

## Data Availability

The datasets used and/or analyzed during the current study are available from the corresponding author on reasonable request.

## Abbreviations

AN: Experimental condition with angry faces
ANOVA: Analysis of variance
BDI: Beck Depression Inventory
BVAQ: Bermond-Vorst Alexithymia Questionnaire
DDF: Difficulties describing feelings (subscale of TAS-20 and TSIA)
DIF: Difficulties identifying feelings (subscale of TAS-20 and TSIA)
DSM-IV: Diagnostic and Statistical Manual of Mental Disorders, fourth edition
EOT: Externally oriented thinking (subscale of TAS-20 and TSIA)
EPI: Echo planar imaging
FE: Experimental condition with fearful faces
(f)MRI: (Functional) magnetic resonance imaging
FWE: Family-wise error
HA: Experimental condition with happy faces
IMG: Imaginal processes (subscale of the TSIA)
MNI: Montreal Neurological Institute
NE: Experimental condition with neutral faces
ROI: Region of interest
SPM8: Statistical Parametric Mapping version 8
STAI: State-Trait Anxiety Inventory
STG: Superior temporal gyrus
TAS-20: 20-Item Toronto Alexithymia Scale
TSIA: Toronto Structured Interview for Alexithymia
WFU: Wake Forest University
