# Supplementary material for: Alexithymia and automatic processing of facial emotions: behavioral and neural findings
Source: BMC Neurosci. 2020 May 29;21:23. doi: 10.1186/s12868-020-00572-6 (PMC7257227; doi:10.1186/s12868-020-00572-6)
Supplement: Supplementary file 1 — Additional file 1: Table S1. Correlations between alexithymia measures (N = 49). [file 12868_2020_572_MOESM1_ESM.docx]

Table S1 Correlations between alexithymia measures (N = 49)

|  | TAS-20 DIF | TAS-20 DDF | TAS-20 EOT | BVAQ Total | BVAQ Identi-fying | BVAQ  Verba-lizing | BVAQ  Analyzing | BVAQ  Emotiona-lizing | BVAQ Fanta-sizing | TSIA Total | TSIA DIF | TSIA DDF | TSIA EOT | TSIA IMP |
| --- | --- | --- | --- | --- | --- | --- | --- | --- | --- | --- | --- | --- | --- | --- |
| TAS-20 Total | .76*** | .84*** | .77*** | .82*** | .62*** | .82*** | .71*** | .53*** | .25 | .43** | .37** | .55*** | .40** | .10 |
| TAS-20 DIF | 1 | .50*** | .34* | .46** | .69*** | .46** | .32* | .29* | -.05 | .14 | .31* | .31* | .07 | -.17 |
| TAS-20 DDF |  | 1 | .49*** | .80*** | .47** | .90*** | .71*** | .44** | .30* | .38** | .30* | .50*** | .33* | .11 |
| TAS-20 EOT |  |  | 1 | .68*** | .33* | .58*** | .65*** | .52*** | .33* | .49*** | .27 | .49*** | .52*** | .28 |
| BVAQ Total |  |  |  | 1 | .58*** | .86*** | .87*** | .73*** | .51*** | .59*** | .44** | .65*** | .54*** | .31* |
| BVAQ Identifying |  |  |  |  | 1 | .49*** | .40** | .33* | -.06 | .21 | .37** | .35* | .12 | -.10 |
| BVAQ  Verbalizing |  |  |  |  |  | 1 | .73** | .49*** | .28 | .46** | .32* | .56*** | .45** | .16 |
| BVAQ  Analyzing |  |  |  |  |  |  | 1 | .64*** | .31* | .47** | .36** | .52*** | .46** | .21 |
| BVAQ  Emotionalizing |  |  |  |  |  |  |  | 1 | .17 | .51*** | .42** | .60*** | .50*** | .16 |
| BVAQ Fantasizing |  |  |  |  |  |  |  |  | 1 | .42** | .12 | .27 | .34* | .61*** |
| TSIA Total |  |  |  |  |  |  |  |  |  | 1 | .79*** | .89*** | .92*** | .71*** |
| TSIA DIF |  |  |  |  |  |  |  |  |  |  | 1 | .81*** | .64*** | .30* |
| TSIA DDF |  |  |  |  |  |  |  |  |  |  |  | 1 | .72*** | .39** |
| TSIA EOT |  |  |  |  |  |  |  |  |  |  |  |  | 1 | .63*** |
| TSIA IMP |  |  |  |  |  |  |  |  |  |  |  |  |  | 1 |

TAS-20 = 20-item Toronto Alexithymia Scale; DIF = Difficulties Identifying Feelings; DDF = Difficulties Describing Feelings; EOT = Externally Oriented Thinking; BVAQ = Bermond-Vorst Alexithymia Questionnaire; TSIA = Toronto Structured Interview for Alexithymia; IMP = Imaginal Processes.

*** *p* < .001, ** *p* < .01, * *p* < .05 (two-tailed).
